# Unmasking Pheochromocytoma, a Rare Case of Diabetic Ketoacidosis as the First Clue

**DOI:** 10.1210/jcemcr/luaf135

**Published:** 2025-06-24

**Authors:** Dina Saba, Mehreen Malik Khan, Joseph An Vu

**Affiliations:** Kaiser Permanente, Santa Clara, CA 95051, USA; Endocrinology, Kaiser Permanente, Mountain View, CA 94041, USA; Endocrinology, Kaiser Permanente, San Jose, CA 95119, USA

**Keywords:** pheochromocytoma, diabetic ketoacidosis (DKA), adrenal mass, adrenalectomy

## Abstract

We report the case of a 62-year-old woman with nausea, vomiting, abdominal pain, polyuria, and polydipsia presenting to the emergency room and found to have new-onset diabetes with diabetic ketoacidosis (DKA). Incidentally discovered bilateral adrenal masses found during evaluation for possible abdominal abscess prompted further investigation, and pheochromocytoma was confirmed through biochemical analysis. She was treated with adrenalectomy, resulting in resolution of diabetes. This case demonstrates a unique presentation of pheochromocytoma with DKA.

## Introduction

Pheochromocytomas (PCCs) are rare catecholamine-secreting tumors of the adrenal medulla, which typically cause refractory episodes of hypertension, headaches, sweating, and palpitations [1]. PCCs can also cause mild hyperglycemia by increasing glucagon release and raising insulin resistance. Here we report a case of PCC with an exceedingly uncommon presentation of diabetic ketoacidosis (DKA), only documented a few times before but clinically significant due to the potential for life-threatening complications [2-4]. This case report aims to highlight the diagnostic challenges and management strategies for such a presentation, emphasizing the role of a thorough workup when adrenal masses are identified incidentally in DKA.

## Case Presentation

A 62-year-old woman with a known history of hypertension for the past 12 years treated with just amlodipine 5 mg, presented to the emergency department (ED) with a 3-day history of nausea, vomiting, abdominal pain, dyspnea, polyuria, and polydipsia. She was found to have severe hyperglycemia with metabolic acidosis and hypertensive emergency with blood pressure of 195/91 mmHg. She had no personal or family history of diabetes. At the time, she did not have diaphoresis, headaches, or palpitations, the classic PCC presentation. She was initially admitted to the intensive care unit (ICU) for the management of DKA and started on a continuous intravenous insulin infusion at a rate of 0.1 units/kg/h, along with aggressive fluid resuscitation. As her acidosis resolved and she demonstrated stable blood glucose control with closure of the anion gap, she was transitioned to subcutaneous insulin therapy with Neutral Protamine Hagedorn insulin (NPH) 27 units twice daily. Throughout her hospitalization, she required higher insulin doses than expected, raising suspicion for an underlying secondary cause of insulin resistance.

## Diagnostic Assessment

During initial septic workup, a computed tomography (CT) scan of the abdomen revealed a 6.6-cm (2.60 in) right adrenal mass with internal hemorrhage and a 2.6-cm (1.02 inches) left adrenal lesion consistent with a lipid rich adenoma. CT scan of the chest additionally noted lobulated right thyroid gland. A thyroid ultrasound confirmed a 4.6 cm (1.81 in) spongiform nodule at the inferior right lobe and a 3.4 cm (1.34 inches) TI-RADS 4 nodule at the left lobe. Fine-needle aspiration of the left inferior thyroid revealed benign cytology. Given the new-onset diabetes, refractory hypertension, and imaging findings, endocrine consultation was obtained, and further biochemical testing was initiated to evaluate the incidental adrenal masses [5]. Initial overnight dexamethasone suppression tests showed elevated cortisol levels at 8 µg/dL (220.7 nmol/L) (normal reference range: <1.8 µg/dL; <49.7 nmol/L). Outpatient workup later showed improved cortisol suppression with overnight dexamethasone, salivary cortisol of <0.03 µg/dL (<0.83 nmol/L) (normal reference range: 0.04-0.56 µg/dL; 1.103-15.45 nmol/L) and normal levels of aldosterone and renin, ruling out Cushing syndrome and hyperaldosteronism. Both 24-hour urine and plasma metanephrines were elevated greater than 8 times the upper limit of normal (Table 1). Glutamic acid decarboxylase-65 antibody testing and islet antigen 2 antibody were negative.

**Table 1.** Lab values from day 1, day 8, and day 10 workup

Hormone tested	Day 1	Day 8	Day 10	Normal range
Epinephrine, 24-hour urine	**157.1 µg/TV** **(857.4 nmol/TV)**		**85.2 µg/TV** **(465.1 nmol/TV)**	0.0–20.9 µg/TV (0.0-114.1 nmol/TV)
Norepinephrine, 24-hour Urine	**101.1 µg/TV** **(597.6 nmol/TV)**		42.4 µg/TV (250.6 nmol/TV)	15.0–80.0 µg/TV (88.7-472.9 nmol/TV)
Metanephrine, 24-hour urine	**3731 µg/24 hours** **(10 292.9 nmol/24 hours)**			≤399 µg/24 hours (≤ 1101.2 nmol/24 hours)
Normetanephrine, 24-hour urine	**1210 µg/24 hours** **(20 370 nmol/24 hours)**			138-521 µg/24 hours (753-2845 nmol/24 hours)
Epinephrine, serum		**204 pg/mL** **(1114 pmol/L)**		≤60.4 pg/mL (≤330 pmol/L)
Norepinephrine, serum		293.5 pg/mL (1736 pmol/L)		177.5-811.2 pg/mL (1050–4800 pmol/L)
Normetanephrine, total, serum		**309 pg/mL** **(1690 pmol/L)**		v≤ 148 pg/mL (≤ 808 pmol/L)
Metanephrines, serum		**609 pg/mL** **(3326 pmol/L)**		≤57 pg/mL (≤ 311 pmol/L)
Metanephrines, total, serum		**918 pg/mL** **(4659 nmol/L)**		≤205 pg/mL (≤ 1039 nmol/L)
Carcinoembryonic antigen	**6400 ng/mL** **(6400 µg/L)**			0.0-5000 ng/mL (0.0-5000 µg/L)
Calcium		9.0 mg/dL (2.25 mmol/L)		8.8–10.5 mg/dL (2.20–2.62 mmol/L)
Parathyroid hormone		91 pg/mL (9.65 pmol/L)		26–115 pg/mL (2.76-12.20 pmol/L)
Calcitonin		<2.0 pg/mL (<0.59 pmol/L)		0.0–5.1 pg/mL (0.0-1.49 pmol/L)
Thyroid stimulating hormone	1.4 mIU/L (1.4 IU/L)			0.4–4.5 mIU/L (0.4–4.5 IU/L)

Abnormal values are shown in bold font. Values in parenthesis are International System of Units.

The patient's clinical history of weight loss, smoking, family history of pancreatic cancer and elevated carbohydrate antigen (CA)19-9 and carcinoembryonic antigen (CEA) levels further complicated the diagnostic picture. However, magnetic resonance imaging (MRI) and dedicated CT scan of the pancreas showed no abnormalities.

## Treatment

**Figure 1.**
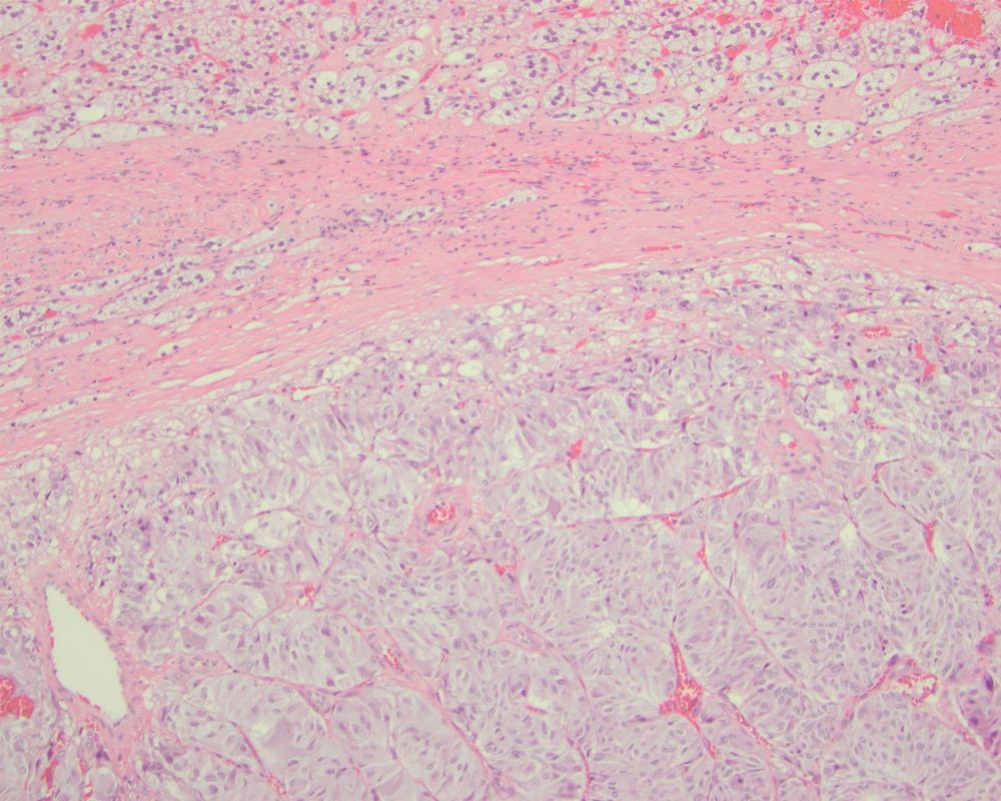
A cellular tumor with areas abutting a normal adrenal gland.

Laparoscopic right adrenalectomy was performed confirming a PCC of the adrenal gland scaled score (PASS) of 9. (Figure 1). Succinate dehydrogenase subunit B (*SDHB*) stain showed no loss. After her confirmed PCC diagnosis, she was referred to our geneticist; however, she declined further genetic testing.

## Outcome and Follow-Up

After resection of the PCC the patient’s hyperglycemia had completely resolved and she had no residual antihyperglycemic requirements. She was left with minimal requirements for hypertension, only 2.5 mg of amlodipine once daily.

## Discussion

PCCs are notorious for causing episodic or sustained hypertension due to excess catecholamine production, which can also induce insulin resistance and mild hyperglycemia. In this rare case, in a patient without a prior diagnosis of diabetes, there was progression to DKA. The presence of a large necrotic adrenal mass, coupled with refractory insulin requirements and elevated catecholamine levels, strongly supported the diagnosis of PCC.

Previous reports of PCC presenting as DKA have primarily described younger patients. Sedhai (2016) reported a 30-year-old man with hypertensive urgency, DKA, and a left adrenal PCC [2], while Hedley (2016) described a 39-year-old woman with similar findings [3]. In both cases, adrenalectomy led to resolution of hyperglycemia and hypertension. Nakamura (2001) documented a 31-year-old woman with DKA and an incidental left adrenal tumor, which was later confirmed as PCC. Unlike these cases, our patient was significantly older and presented with bilateral adrenal abnormalities, although only the right-sided lesion was functional.

A notable feature of this case is the absence of the classic PCC triad of headache, palpitations, and sweating [6]. This underscores the need for a high index of suspicion for PCC in patients with DKA in the setting of adrenal masses or refractory hypertension. Additionally, this patient had longstanding hypertension managed with amlodipine, which may have masked the hypertensive episodes typically seen in PCC, complicating the clinical picture [7].

The link between PCC and DKA is primarily driven by the metabolic effects of excess catecholamines, which promote gluconeogenesis, inhibit insulin secretion, and increases lipolysis, all of which can precipitate ketoacidosis. Unlike DKA due to type 1 diabetes mellitus (T1DM), where absolute insulin deficiency is the hallmark, PCC-induced DKA is driven by extreme catecholamine excess causing profound insulin resistance, impaired insulin secretion, and enhanced counter-regulatory hormone activity for a long duration. In this patient, the levels of epinephrine 8 times the upper limit of normal from a large necrotic mass resulted in lipolysis, gluconeogenesis, and suppression of insulin from the pancreas. The degree of catecholamine secretion was high enough to override residual insulin action, tipping the metabolic balance from isolated hyperglycemia to full ketoacidosis. The combination of increased lipolysis, glycogenolysis, and proteolysis, particularly in the setting of critical illness or prolonged fasting, created a metabolic environment conducive to ketone production and acidosis. Additionally, impaired peripheral glucose utilization contributed to the development of DKA rather than the more typical hyperglycemia seen with PCC.

Ordinarily, postoperative management requires careful control of blood pressure and monitoring of blood glucose to avoid hypoglycemia. In this case no postoperative hypoglycemia was noted, and blood pressure had improved.

## Learning Points

- It is important to consider pheochromocytoma as a potential underlying cause of DKA, particularly in patients with adrenal masses or unexplained hypertension.
- Early diagnosis and surgical intervention are critical to prevent the life-threatening sequelae associated with this rare endocrine tumor.
- It is essential for clinicians to maintain a high index of suspicion for secondary causes of DKA when typical risk factors are absent or when adrenal pathology is detected incidentally.

## Contributors

All authors made individual contributions to authorship. M.K. and J.V. participated in the diagnosis and management of the patient. D.S. was responsible for the literature review and manuscript submission. All authors reviewed and approved the final draft.

## Data Availability

Original data generated and analyzed during this study are included in this published article.

## Abbreviations

CT: computed tomography
DKA: diabetic ketoacidosis
PCC: pheochromocytoma
